# An association between heart rate variability and pediatric obstructive sleep apnea

**DOI:** 10.1186/s13052-024-01576-2

**Published:** 2024-03-18

**Authors:** Nuntigar Sonsuwan, Krittika Houngsuwannakorn, Nipon Chattipakorn, Kittisak Sawanyawisuth

**Affiliations:** 1 Department of Otolaryngology Faculty of Medicine, Chiang Mai University, Chiang Mai, Thailand; 2 Sansai Hospital, Chiang Mai, Thailand; 3 Cardiac Electrophysiology Research and Training Center, Faculty of Medicine, Chiang Mai University, Chiang Mai, Thailand; 4 Department of Medicine, Faculty of Medicine, Khon Kaen University, 123 Mitraparp Road, 40002 Khon Kaen, Thailand

**Keywords:** Standard deviation of all normal interval, High frequency, Low frequency, Apnea-hypopnea index, Pediatric obstructive sleep apnea

## Abstract

**Background:**

There are different findings on heart rate variability (HRV) and pediatric obstructive sleep apnea (pOSA) by an overnight HRV or a 1-hr HRV. However, there is limited data of HRV and pOSA diagnosis by using a 24-h HRV test. This study aimed to evaluate if HRV had potential for OSA diagnosis by using a 24-h HRV test.

**Methods:**

This was a prospective study included children age between 5 and 15 years old, presenting with snoring, underwent polysomnography and a 24-h Holter monitoring. Predictors for pOSA diagnosis were analyzed using logistic regression analysis.

**Results:**

During the study period, there were 81 pediatric patients met the study criteria. Of those, 65 patients (80.25%) were diagnosed as OSA. There were three factors were independently associated with OSA: standard deviation of all normal interval (SDNN), high frequency (HF), and low frequency (LF). The adjusted odds ratios of these factors were 0.949 (95% confidence interval 0.913, 0.985), 0.786 (95% confidence interval 0.624, 0.989), and 1.356 (95% confidence interval 1.075, 1.709).

**Conclusions:**

HRV parameters including SDNN, HF, and LF were associated with pOSA diagnosis in children by using the 24-h Holter monitoring.

## Background

Pediatric obstructive sleep apnea (pOSA) has an average prevalence rate of OSA from 1 to 4% [1]. The pOSA prevalence rate is higher in children with suspected symptoms of OSA such as snoring or in children with co-morbid diseases such as asthma. The prevalence rates of pOSA were reported as 44% in children with snoring, 46% in obese children and 29.6% in children with asthma [2–4]. Similary to adult patients with OSA, children with pOSA are also at risk for several consequences from OSA such as daytime sleepiness, poor quality of life, impaired neurocognitive function, and cardiovascular diseases [5–10]. Children with pOSA had an elevation of blood pressure during awake and while sleep compared with control by 10–15 mmHg [11].

Heart rate variability (HRV) is an indicator of cardiac autonomic activity and also an indicator of future cardiovascular diseases. Compared with the highest tertile of HRV, the lowest HRV tertile group had higher lifetime cardiovascular risks in both men (49% vs. 45%) and women (38% vs. 30%) [12]. A systematic review found that patients with OSA may have lower vagal tone and higher sympathetic activity [13]. In children with pOSA had higher high frequency than control in all stages of sleep such as 1836 vs. 1456 in REM sleep (*p* < 0.01) [14]. A previous study found that seven children with pOSA had different HRV than seven children with primary snoring in beat-to-beat variations in slow, intermediate, and fast heart rates [15]. Other two studies also showed different low/high frequency ratio between children with pOSA and primary snoring but with overnight HRV or a 1-hr HRV [16, 17].

In adults with OSA, HRV is a reliable tool for OSA diagnosis with an accuracy rate of 94.12% [18]. For pOSA, HRV is a potential tool for OSA diagnosis and OSA resolution [16, 19–22]. Children with mild pOSA had significantly higher low frequency/high frequency (LF/HF) than primary snorers (1.3 vs. 1.0; *p* < 0.001) [22]. Another study also found that children with moderate pOSA had significant reduction in parasympathetic in rapid eye movement sleep than those without pOSA: HF 4.49 vs. 5.80 ms^2^; *p* < 0.05 [20]. Even though several studies showed abnormal HRV in pOSA, there is limited data on using HRV as a diagnostic tool for pOSA. Therefore, the primary objective of this study was to evaluate if HRV could lead to pOSA diagnosis. If so, which factors of HRV could be predictors of pOSA.

## Materials and methods

This was a prospective study conducted at Department of Otorhinolaryngology, Faculty of Medicine, Chiang Mai University, Chiang Mai, Thailand. The inclusion criteria were children age between 5 and 15 years old, presenting with snoring, underwent polysomnography and Holter monitoring. Those who were unable to perform either polysomnography or Holter monitoring, had active arrhythmia, cardiovascular diseases, congenital/ medical/ or chronic health conditions including mental retardation, cerebral palsy, or autism, or did not agree for study participation were excluded. The study period was between January and August 2016.

Eligible children were evaluated for baseline characteristics, physical signs, an overnight polysomnography, and a 24-h Holter monitoring. Baseline characteristics included age, sex, body mass index, and co-morbid diseases. Grading of adenoid gland and tonsil were evaluated. Tonsil size was assessed by oropharyngeal examination and graded as grade 0 to 4, while adenoid gland was examined by a flexible nasopharyngoscope and graded as grade 1 to 4 [23, 24].

An overnight polysomnography was performed by Somnocheck ® (Weinmann, Hamburg, Germany) for at least 6 h. Diagnosis of OSA was made if an apnea-hypopnea index (AHI) of 1 event/hr or over, while an AHI of 1 or lower was classified as primary snoring [5]. Scoring system for OSA was performed according to the American Academy of Sleep Medicine (AASM) scoring manual [25].

A 24-h Holter monitoring was underwent by the GE model Seer Light Extend 24 for HRV evaluation (Seer Light, GE Medical Systems, USA) and reported as time domain in msec (ms) and frequency domain. The time domain comprised of standard deviation of all normal interval (SDNN), standard deviation of the normal interval means of every 5 min (SDANN), an average of the standard deviations of all R-R intervals for all 5-min segments in the 24-h recording (ASDNN), root-mean square of difference between adjacent normal interval (rMSDNN). The frequency domain had three variables including LF (ms^2^), HF (ms^2^), and LF/HF ratio. The parameters of HRV were analyzed and reported by the computer software of GE Healthcare’s MARS Holter Analysis System as previously reported elsewhere [26]. The primary endpoint of the study was the diagnosis of pOSA.

Statistical analyses. Eligible patients were categorized into two groups: primary snoring and OSA. Descriptive statistics were used to calculate differences between the two groups. Predictors for pOSA diagnosis were analyzed using logistic regression analysis. Univariate logistic analysis was used to calculate the unadjusted odds ratio with 95% confidence interval and *p* value for each factor. Factors with a p value less than 0.05 by univariate logistic regression analysis were subsequently subjected to stepwise, multivariate logistic regression analysis. The final model was tested for goodness of fit using the Hosmer-Lemeshow method. A numerical predictor for OSA as an appropriate diagnostic cutoff point was computed and reported its sensitivity and specificity. All statistical analyses were performed using STATA version 10.1 (College Station, Texas, USA).

## Results

During the study period, there were 81 pediatric patients met the study criteria. Of those, 65 patients (80.25%) were diagnosed as OSA, while 16 patients were primary snoring. Baseline characteristics, co-morbid diseases, and physical examination were comparable between those with and without OSA (Table 1). The AHI was significantly higher in the OSA group than the Non-OSA group (6 vs. 0 events/h; *p* < 0.001).

**Table 1:** Baseline characters of children with primary snoring or obstructive sleep apnea (OSA) who participated in the heart rate variability study

Factors	Primary snoring *n* = 16	OSA *n* = 65	***P***-value
Age, year	8 (5–11)	8 (5–15)	0.657
Male sex	12 (75.00)	48 (73.85)	0.224
Co-morbid diseases			
Pulmonary hypertension	0	2 (3.08)	0.999
Hypertension	0	2 (3.08)	0.999
Stroke	0	1 (1.54)	0.999
ADHD	1 (6.25)	5 (7.69)	0.999
Delayed development	0	3 (4.62)	0.999
Weight, kg	34.85 (18.00-70.40)	35.50 (15.80-106.8)	0.384
Height, cm	130 (108–155)	129 (97–179)	0.678
Body mass index, kg/m^2^	19.10 (14.00-32.30)	22.32 (12.00–40.00)	0.345
Tonsil grading			0.363
0	1 (6.25)	15 (23.44)	
1	1 (6.25)	1 (1.56)	
2	5 (31.25)	15 (23.44)	
3	5 (31.25)	21 (32.81)	
4	4 (25.00)	12 (18.75)	
Adenoid grading			0.687
1	0	7 (10.94)	
2	8 (50.00)	27 (42.19)	
3	7 (43.75)	23 (35.94)	
4	1 (6.25)	7 (10.94)	
Adenotonsillectomy	1 (6.25)	11 (16.92)	0.443
AHI, events/h	0 (0–1)	6 (1.7–23.6)	< 0.001

Note: Data presented as median (range) or number (percentage); ADHD: Attention-deficit/hyperactivity disorder; AHI: apnea-hypopnea index

For HRV parameters, there was no statistical significant difference between the OSA and primary snoring group (Table 2). The OSA group had slightly shorter SDANN (94 vs. 104 ms; *p* = 0.056) and higher LF/HF ratio (1.28 vs. 1.20) than the primary snoring group. There were four factors remaining in the final model predictive of OSA (Table 3). Of those, three factors were independently associated with OSA: SDNN, HF, and LF. The adjusted odds ratios of these factors were 0.949 (95% confidence interval 0.913, 0.985), 0.786 (95% confidence interval 0.624, 0.989), and 1.356 (95% confidence interval 1.075, 1.709). The Hosmer-Lemeshow Chi square of the final model was 13.56 (*p* = 0.094). SDNN of 138 ms or lower and HF of 25.38 ms^2^ or lower yielded a sensitivity of 80.00% for both factors and specificity of 31.25% for SDNN and 12.50% for HF. LF of 17.36 ms^2^ or higher had a sensitivity of 80.00% and specificity of 6.25%, while LF of 25.89 ms^2^ or higher increased a specificity to 81.25% with a sensitivity of 43.08%.

**Table 2:** Heart rate variability parameters of children with primary snoring or obstructive sleep apnea (OSA).

Factors	Primary snoring *n* = 16	OSA *n* = 65	***P***-value
Time domain			
SDNN, ms	115 (90–194)	111 (40–186)	0.165
SDANN, ms	104 (71–184)	94 (35–178)	0.056
ASDNN, ms	57 (40–121)	61 (17–100)	0.721
rMSSD, ms	38 (28–73)	38 (9–64)	0.830
Frequency domain			
LF, ms^2^	22.33 (13.52–45.81)	23.82 (5.81–43.09)	0.581
HF, ms^2^	18.46 (12.20-54.51)	19.79 (2.05–36.96)	0.896
LF/HF ratio	1.20 (0.81–1.71)	1.28 (0.87–2.83)	0.088

Note: Data presented as median (range) or number (percentage); SDNN: standard deviation of all normal interval; SDANN: standard deviation of the normal interval means of every 5 min; ASDNN: average of the standard deviations of all R-R intervals for all 5-min segments in the 24-h recording; rMSSD: root-mean square of difference between adjacent normal interval; LF: low frequency; HF: high frequency

**Table 3:** Factors associated with obstructive sleep apnea in children underwent heart rate variability study

Factors	Unadjusted odds ratio (95% confidence interval)	Adjusted odds ratio (95% confidence interval)
SDNN, ms	0.984 (0.967, 1.001)	0.949 (0.913, 0.985)
rMSSD, ms	0.985 (0.942, 1.031)	1.093 (0.952, 1.255)
LF, ms^2^	1.016 (0.949, 1.087)	1.356 (1.075, 1.709)
HF, ms^2^	0.979 (0.919, 1.044)	0.786 (0.624, 0.989)

Note: Factors in the model included age, sex, body mass index, adenoid grading, tonsil grading, adenotonsillectomy, and apnea-hypopnea index; SDNN: standard deviation of all normal interval; rMSSD: root-mean square of difference between adjacent normal interval; LF: low frequency; HF: high frequency

## Discussion

Even though there are several predictors of pOSA, this study found that HRV parameters both time and frequency domain were independently associated with pOSA compared with age, sex, body mass index, and adenotonsillar abnormalities (Table 3) [27, 28].

As previously reported, SDNN was negatively associated with OSA in adult patients with OSA [29]. The SDNN levels suggestive for OSA between adults and children were slightly different (177 vs. 138 ms). This correlation may be explained from OSA induced HRV [30, 31]. Another difference between this study and the adult study was that sex was not a predictor for pOSA in this study, while sex was also independently associated with being OSA in adult patients with OSA [29]. These results may imply that SDNN may be a stronger predictor for pOSA than sex or even AHI.

HF, an indicator of parasympathetic system, was negatively associated with pOSA. Children with pOSA had decreasing parasympathetic activity compared with non-OSA children [32]. HF was significantly lower in children with pOSA compared with non-OSA children (*p* = 0.007). These results may be explained by negative correlation between AHI and HF (rho = -0.36. *p* = 0.021). Another study also found that HF was lower in only rapid eye movement (REM) sleep [16].

Unlike HF, LF may be an indicator of both sympathetic and parasympathetic activities. A previous study found that patients with severe OSA had an increasing in LF than moderate OSA group (5292 vs. 3135; *p* = 0.002) [33]. Additionally, patients with OSA tended to have more adrenergic dysfunction than non-OSA patients (80% vs. 30%; *p* < 0.05) [34]. These results may indicate that patients with OSA may have autonomic dysfunction resulting in increasing LF and related to OSA diagnosis particularly those with severe OSA (Tables 2 and 3).

Even though this study found that the 24-h HRV monitoring may be useful for OSA diagnosis in children, there are some limitations in this study. First, children participated in this study needed to wear the Holter monitoring for a whole day. Second, the control group for this study was primary snorer, not normal control as the study conducted in the snoring clinic. However, there were still independent factors for OSA as shown in Table 3. Further studies with normal control may be needed. Finally, this study population was limited to 15 years and some related factors are not studied [35–40].

## Conclusions

HRV parameters including SDNN, HF, and LF were associated with pOSA diagnosis in children by using the 24-h Holter monitoring.

## Data Availability

The data used to support the fndings of this study are available from the corresponding author upon request.

## Abbreviations

AHI: Apnea-hypopnea index
ASDNN: An average of the standard deviations of all R-R intervals for all 5-min segments in the 24-h recording
HF: High frequency
HRV: Heart rate variability
LF: Low frequency
pOSA: Pediatric obstructive sleep apnea
REM: Rapid eye movement
rMSDNN: Root-mean square of difference between adjacent normal interval
SDNN: Standard deviation of all normal interval
SDANN: Standard deviation of the normal interval means of every 5 minutes
